# Wind Tunnel Measurements for Flutter of a Long-Afterbody Bridge Deck

**DOI:** 10.3390/s17020335

**Published:** 2017-02-09

**Authors:** Zeng-Shun Chen, Cheng Zhang, Xu Wang, Cun-Ming Ma

**Affiliations:** 1 State Key Laboratory Breeding Base of Mountain Bridge and Tunnel Engineering, Chongqing Jiaotong University, Chongqing 400074, China; zchenba@connect.ust.hk; 2 Department of Civil and Environmental Engineering, The Hong Kong University of Science and Technology, Kowloon, Hong Kong, China; 3 Shenzhen Bridge Design & Research Institute Co., Ltd., Shenzhen 518052, China; 4 School of Civil Engineering, Southwest Jiaotong University, Chengdu 610031, China; mcm@home.swjtu.edu.cn

**Keywords:** aerodynamic force, flutter derivatives, critical flutter wind speed, long-afterbody bridge deck

## Abstract

Bridges are an important component of transportation. Flutter is a self-excited, large amplitude vibration, which may lead to collapse of bridges. It must be understood and avoided. This paper takes the Jianghai Channel Bridge, which is a significant part of the Hong Kong-Zhuhai-Macao Bridge, as an example to investigate the flutter of the bridge deck. Firstly, aerodynamic force models for flutter of bridges were introduced. Then, wind tunnel tests of the bridge deck during the construction and the operation stages, under different wind attack angles and wind velocities, were carried out using a high frequency base balance (HFBB) system and laser displacement sensors. From the tests, the static aerodynamic forces and flutter derivatives of the bridge deck were observed. Correspondingly, the critical flutter wind speeds of the bridge deck were determined based on the derivatives, and they are compared with the directly measured flutter speeds. Results show that the observed derivatives are reasonable and applicable. Furthermore, the critical wind speeds in the operation stage is smaller than those in the construction stage. Besides, the flutter instabilities of the bridge in the construction and the operation stages are good. This study helps guarantee the design and the construction of the Jianghai Channel Bridge, and advances the understanding of flutter of long afterbody bridge decks.

## 1. Introduction

With the ever-growing span-length of bridges, the action of wind on the long-span bridges becomes more important than before. Wind tunnel test is widely accepted to evaluate the action of wind on structures [1,2]. The aerodynamic instability of bridges is primarily concerned in the design of long-span bridges, especially for those located in regions prone to be affected by wind [3]. Flutter is one of the aerodynamic instabilities, which is a phenomenon of self-excited vibration. It takes place when a structure is exposed to wind velocities above a certain critical value which can be evaluated experimentally or theoretically [4]. Above the critical value, flutter may cause a structure to oscillate continuously with increasing amplitude until the structure collapses. Therefore, flutter must be studied and avoided during the construction and the operation stages of a structure [5]. 

After the failure of the Tacoma Narrow Bridge, many efforts have been made for evaluating flutter of bridges [6,7,8,9,10]. Though the consideration of flutter for the design of long-span bridges has a long history, new challenges and problems were raised in terms of the geometrical configurations of bridges [11,12]. To theoretically evaluate flutter of bridges, the key problem is to identify the flutter derivatives of bridges. An identification method was first proposed by Scanlan [13]. In the method, a spring-suspended sectional model was tested in a wind tunnel and the free decay vibration signals were used. Afterwards, many efforts have been made by researchers to simplify the identification method [14,15,16,17]. After determination of the flutter derivatives of a bridge deck, the corresponding critical flutter wind speed can be evaluated by using appropriate methods [5,18]. All the above studies about flutter derivatives identification and critical wind speed calculation have significantly helped evaluation of flutter of structures. Despite the progress achieved, flutter of bridge decks, especially those with irregular geometrical configurations (i.e., slotted or long-afterbody geometries), should be well investigated. 

This study focused on flutter of the Jianghai Channel Bridge which is an important component of the Hong Kong-Zhuhai-Macao bridge and whose bridge deck is characterized by long afterbody (B/D = 8.63, where B and D are the width and the depth of the bridge deck, respectively). Aerodynamic force models for flutter of bridges were introduced. Then, wind tunnel tests of the bridge deck during the construction and the operation stages of the bridge were carried out. From the tests, flutter derivatives were identified and compared with theoretically evaluated results. Correspondingly, the critical wind speeds of the flutter of the bridge deck were determined based on the identified flutter derivatives. This study helps guarantee the design and the erection of the Jianghai Channel Bridge, and advances the understanding of flutter of long after body bridge decks.

## 2. Aerodynamic Force Model 

Based on the potential energy principle proposed by Theodorson [19], the differential equations governing the motion of a bridge deck model under the action of wind can be directly given as follows:

(1a) $$m(\ddot{h}+2\zeta_{h}\omega_{h}\dot{h}+\omega_{h}^{2}h)=L_{se}$$

(1b) $$I(\ddot{\alpha }+2\zeta_{\alpha }\omega_{\alpha }\dot{\alpha }+\omega_{\alpha }^{2}\alpha )=M_{se}$$

(1c) $$m(\ddot{p}+2\zeta_{p}\omega_{p}\dot{p}+\omega_{p}^{2}p)=P_{se}$$

where m and I are the model mass and mass inertia moment per unit length, respectively; and $\zeta_{h}$, $\zeta_{\alpha }$, and $\zeta_{p}$ are the mechanical damping ratios in bending, torsion and swaying, respectively. Correspondingly, $\omega_{h}$, $\omega_{\alpha }$, and $\omega_{p}$ are the natural frequencies; and $L_{se}$, $M_{se}$, and $P_{se}$ are the aerodynamic self-exited forces and moments.

The aerodynamic self-excited forces and moments can be directly given by [20]

(2a) $$L_{se}=\rho U^{2}B[KH_{1}^{*}\frac{\dot{h}}{U}+KH_{2}^{*}\frac{B\dot{\alpha }}{U}+K^{2}H_{3}^{*}\alpha +K^{2}H_{4}^{*}\frac{h}{B}+KH_{5}^{*}\frac{\dot{P}}{U}+K^{2}H_{6}^{*}\frac{P}{B}],$$

(2b) $$M_{se}=\rho U^{2}B[KA_{1}^{*}\frac{\dot{h}}{U}+KA_{2}^{*}\frac{B\dot{\alpha }}{U}+K^{2}A_{3}^{*}\alpha +K^{2}A_{4}^{*}\frac{h}{B}+KA_{5}^{*}\frac{\dot{P}}{U}+K^{2}A_{6}^{*}\frac{P}{B}],$$

(2c) $$P_{se}=\rho U^{2}B[KP_{1}^{*}\frac{\dot{h}}{U}+KP_{2}^{*}\frac{B\dot{\alpha }}{U}+K^{2}P_{3}^{*}\alpha +K^{2}P_{4}^{*}\frac{h}{B}+KP_{5}^{*}\frac{\dot{P}}{U}+K^{2}P_{6}^{*}\frac{P}{B}],$$

where K is reduced frequency, which is defined as K=Bω/U; ρ is air density; B is the width of the sectional bridge deck model; U is the mean velocity of the coming wind; and $H_{i}^{*},A_{i}^{*},P_{i}^{*}(i=1,...,6)$ are the flutter derivations of the deck model.

An optimization model involving twin undetermined parameters for determining the critical flutter wind speeds of the deck model proposed by Xu et al. [21], is briefly introduced as follows.

Let,

(3) $$s=Ut/B,K_{h}=B\omega_{h}/U,K_{p}=B\omega_{p}/U,K_{\alpha }=B\omega_{\alpha }/U$$

(4) $$h=h_{0}\cdot e^{iKs},p=p_{0}\cdot e^{iKs},\alpha =\alpha_{0}\cdot e^{iKs}$$

Substituting Equations (3) and (4) into Equations (1) and (2), we have

(5a) $$\begin{array}{l} -\frac{h_{0}}{B}\cdot K^{2}+2\zeta_{h}KK_{h}\frac{h_{0}}{B}\cdot (i)+K_{h}^{2}\frac{h_{0}}{B}\\ =\frac{\rho B^{2}}{m}[K^{2}H_{1}^{*}\frac{h_{0}}{B}\cdot (i)+K^{2}H_{2}^{*}\alpha_{0}\cdot (i)+K^{2}H_{3}^{*}\alpha_{0}+K^{2}H_{4}^{*}\frac{h_{0}}{B}+K^{2}H_{5}^{*}\frac{P_{0}}{B}\cdot (i)+K^{2}H_{6}^{*}\frac{P_{0}}{B}] \end{array}$$

(5b) $$\begin{array}{l} -\alpha_{0}\cdot K^{2}+2\zeta_{\alpha }KK_{\alpha }\alpha_{0}\cdot (i)+K_{\alpha }^{2}\alpha_{0}\\ =\frac{\rho B^{4}}{I}[K^{2}A_{1}^{*}\frac{h_{0}}{B}\cdot (i)+K^{2}A_{2}^{*}\alpha_{0}\cdot (i)+K^{2}A_{3}^{*}\alpha_{0}+K^{2}A_{4}^{*}\frac{h_{0}}{B}+K^{2}A_{5}^{*}\frac{P_{0}}{B}\cdot (i)+K^{2}A_{6}^{*}\frac{P_{0}}{B}] \end{array}$$

(5c) $$\begin{array}{l} -\frac{p_{0}}{B}\cdot K^{2}+2\zeta_{p}KK_{p}\frac{p_{0}}{B}\cdot (i)+K_{p}^{2}\frac{p_{0}}{B}\\ =\frac{\rho B^{2}}{m}[K^{2}P_{1}^{*}\frac{h_{0}}{B}\cdot (i)+K^{2}P_{2}^{*}\alpha_{0}\cdot (i)+K^{2}P_{3}^{*}\alpha_{0}+K^{2}P_{4}^{*}\frac{h_{0}}{B}+K^{2}P_{5}^{*}\frac{P_{0}}{B}\cdot (i)+K^{2}P_{6}^{*}\frac{P_{0}}{B}] \end{array}$$

Equation (5) can be simplified as

(6a) $$A_{11}\frac{h_{0}}{B}+A_{12}\frac{P_{0}}{B}+A_{13}\alpha_{0}=0$$

(6b) $$A_{21}\frac{h_{0}}{B}+A_{22}\frac{P_{0}}{B}+A_{23}\alpha_{0}=0$$

(6c) $$A_{31}\frac{h_{0}}{B}+A_{32}\frac{P_{0}}{B}+A_{33}\alpha_{0}=0$$

or

(7) $$\left|\begin{array}{ccc} A_{11} & A_{12} & A_{13}\\ A_{21} & A_{22} & A_{23}\\ A_{31} & A_{32} & A_{33} \end{array}\right|=0$$

where

(8a) $$A_{11}=[-1+{\left(\frac{1}{X}\right)}^{2}-\frac{\rho B^{2}}{m}H_{4}^{*}]+[2\zeta_{h}\frac{1}{X}-\frac{\rho B^{2}}{m}H_{1}^{*}]\cdot (i)$$

(8b) $$A_{21}=-\frac{\rho B^{2}}{m}P_{4}^{*}-\frac{\rho B^{2}}{m}P_{1}^{*}\cdot (i)$$

(8c) $$A_{31}=-\frac{\rho B^{4}}{I}A_{4}^{*}-\frac{\rho B^{4}}{I}A_{1}^{*}\cdot (i)$$

(9a) $$A_{12}=-\frac{\rho B^{4}}{m}H_{6}^{*}-\frac{\rho B^{4}}{m}H_{5}^{*}\cdot (i)$$

(9b) $$A_{22}=[-1+{\left(\frac{\omega_{p}}{\omega_{h}}\right)}^{2}{\left(\frac{1}{X}\right)}^{2}-\frac{\rho B^{2}}{m}P_{6}^{*}]+[2\zeta_{P}\frac{\omega_{p}}{\omega_{h}}\frac{1}{X}-\frac{\rho B^{2}}{m}P_{5}^{*}]\cdot (i)$$

(9c) $$A_{32}=-\frac{\rho B^{4}}{I}A_{6}^{*}-\frac{\rho B^{4}}{I}A_{5}^{*}\cdot (i)$$

(10a) $$A_{13}=-\frac{\rho B^{2}}{m}H_{3}^{*}-\frac{\rho B^{2}}{m}H_{2}^{*}\cdot (i)$$

(10b) $$A_{23}=-\frac{\rho B^{2}}{m}P_{3}^{*}-\frac{\rho B^{2}}{m}P_{2}^{*}\cdot (i)$$

(10c) $$A_{33}=[-1+{\left(\frac{\omega_{\alpha }}{\omega_{h}}\right)}^{2}{\left(\frac{1}{X}\right)}^{2}-\frac{\rho B^{4}}{I}A_{3}^{*}]+[2\zeta_{\alpha }\frac{\omega_{\alpha }}{\omega_{h}}\frac{1}{X}-\frac{\rho B^{4}}{I}A_{2}^{*}]\cdot (i)$$

(11) $$X=\omega /\omega_{h}$$

After determination of flutter derivatives, using a searching critical flutter wind speed method [18,21], the critical flutter wind speed of the deck model can be determined. It should be noted that, usually, the flutter of a long-span bridge in swaying is slight, and therefore only flutter derivatives in bending and torsion are concerned [17,18]. 

## 3. Wind Tunnel Test

Wind tunnel tests were performed to determine flutter derivatives and corresponding critical wind speeds of the bridge deck of the Jianghai Channel Bridge. Static wind loads which can be utilized for evaluating the performance of the bridge were also observed. 

### 3.1. Bridge Overview

**Figure 1:**
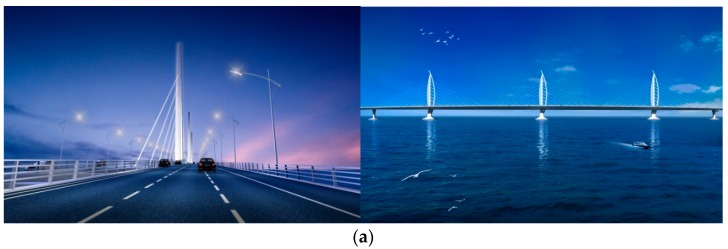

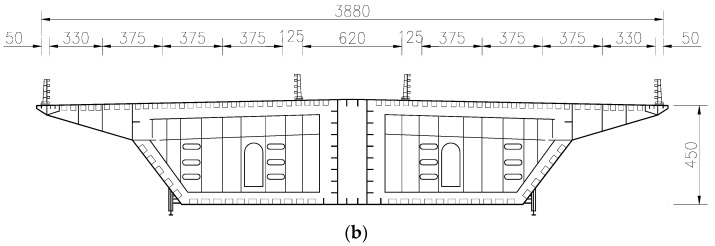
The Jianghai Channel Bridge: (**a**) overview; and (**b**) dimensions of the bridge deck (cm).

The Hong Kong-Zhuhai-Macao Bridge is an important connection of Hong Kong, Zhuhai and Macao. The total length of the bridge is 29.6 km. It is located at the tropical monsoon climate region of the South Asia, which is frequently affected by disastrous weather and strong wind. The Jianghai Channel Bridge (Figure 1) is one of important components of the Hong Kong-Zhuhai-Macao Bridge. The overall length of the Jianghai Channel Bridge is 994 m and the span arrangement is 110 + 129 + 258 + 258 + 129 + 110 m. The bridge girder is a steel box section with a width of 38.8 m (B) and a height of 4.5 m (D). The bridge deck is characterized by long afterbody with B/D = 8.63 (Figure 1). The bridge tower is designed as “Dolphin” exterior and constructed with steel. Its height is 113.756 m. Due to the irregular geometrical configuration of the bridge (i.e., long afterbody), wind action on the bridge during the construction and the operation stages should be well acquired.

### 3.2. Static Force Measurement

**Figure 2:**
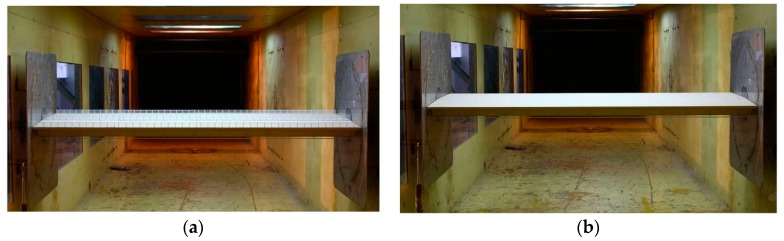
The test models for force measurements: (**a**) the test model in the operation stage; and (**b**) the test model in the construction stage.

The wind tunnel test for the static force measurement of the bridge deck was carried out in the high wind speed test section of the XNJD-1 wind tunnel at the Southwest Jiaotong University in Chengdu, China. The maximum wind speed is 45 m/s and the minimum is 0.5 m/s. The wind profile is determined from the on-site measured parameters and it is directly given as $U_{Z}/U_{10}={\left(Z/10\right)}^{0.098}$. Two bridge deck models, which are used for simulating the bridge decks during the construction and the operation stages, were tested (Figure 2). During the operation stage, accessories were installed on the bridge deck (i.e., crash barrier, hand banister, etc.), but not during the construction stage. The dimensions of the bridge decks in both stages are the same: 69.2 m × 38.8 m (width) × 4.5 m (height). The scale ratios between the prototype and the tests model are 1:50. Thus, the dimensions of the test models are 2.095 m (length) × 0.776 m (width) × 0.09 m (height). 

**Figure 3:**
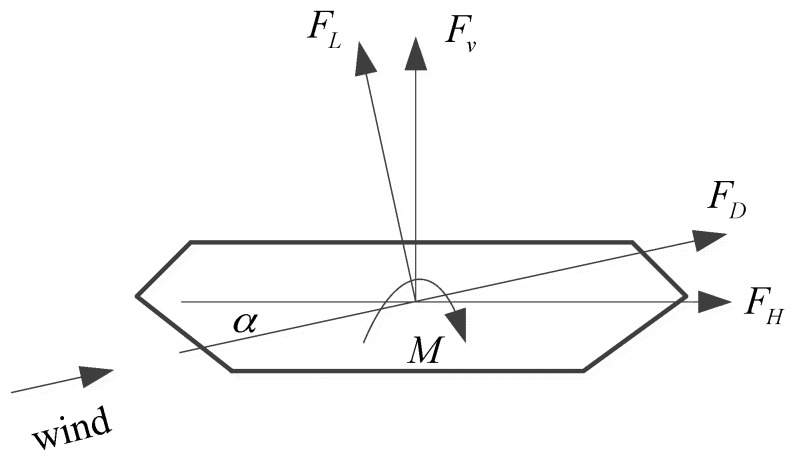
Definitions of aerodynamic force coefficients: drag, lift and moment force.

**Figure 4:**
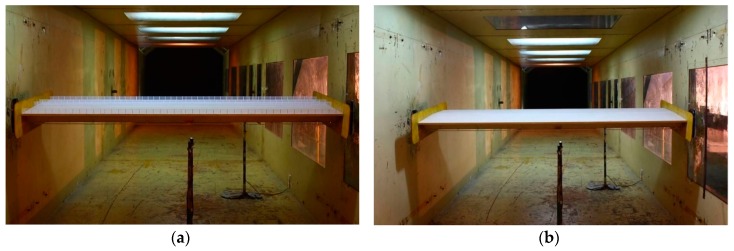
The test models for response measurements: (**a**) the test model in the operation stage; and (**b**) the test model in the construction stage.

To evaluate wind loads of structures, a synchronous multi-pressure sensing system (SMPSS) or a high frequency base balance (HFBB) system can be utilized. In the SMPSS test, the wind loads are evaluated from the observed pressures [22]. In the HFBB test, the base force in different wind directions can be directly observed. In this study, the drag, lift and moment force coefficients were observed by using test HFBB test. The design loads of the balance are 50 kgf, 120 kgf and 12 kgf in drag, lift and motion directions, respectively. The test wind speed is fixed at 18 m/s. The wind attack angle α (Figure 3) varied from −12° to 12°. In Figure 4, $F_{H}$, $F_{V}$ and M are drag, lift and lift-moment forces in wind coordinate axis, respectively. $F_{D}$ and $F_{L}$ are drag and lift forces in body coordinate axis, respectively. 

### 3.3. Flutter Derivatives Test

To obtain flutter derivatives and evaluate the flutter of the bridge deck, free vibration wind tunnel tests were performed in the same wind tunnel. In order to obtain the flutter derivatives of the models at high reduced wind speeds, the mass of the models was adjusted to a high level, 18.7 kg. Then, the flutter derivatives of the models under different wind attack angles ($\alpha =+5^{o},+3^{o},0^{o},-3^{o},-5^{o}$) were observed. Using the derivatives, the critical flutter wind speeds can be determined.

### 3.4. Aeroelastic Test for Flutter Measurement

Another way to evaluate the flutter of the bridge deck is aeroelastic test. From the test, the critical wind speeds of the bridge deck can be directly measured. The test was performed in the same wind tunnel. The wind profile and the dimensions of the models were also the same with that in Section 3.1 (Figure 4). 

The models were suspended on supports by eight springs (Figure 4), and the models could vibrate in vertical and torsional directions under the action of wind. In the test, the ratios of the dynamic parameters (i.e., mass, frequency, and damping ratio) of the models should be consistent with the prototype (Table 1). From Table 1, the maximum difference between the simulated value and required value was 6.1%, and others were within 1%. This suggests that the tests were well simulated and could be performed for evaluating the flutter of the prototype. 

During the test, responses could be measured from different ways, i.e., strain gauges [23], motion-capture cameras [24], etc. In this study, responses of the models under different wind attack angles ($\alpha =+5^{o},+3^{o},0^{o},-3^{o},-5^{o}$) were observed using laser displacement sensors (Micro-Epsilon optoNCDT1401, Micro-Epsilon, Ortenburg, Germany), which are installed below the models (Figure 3).

## 4. Result and Discussion

### 4.1. Aerodynamic Force

The static wind force of the bridge deck is important for evaluation wind actions on the bridge, and it can be utilized to evaluate aeroelastic performance of the deck. The expressions of drag, lift and moment force coefficients (Figure 3) of the test models are given by

(12) $$C_{D}(\alpha )=\frac{F_{D}(\alpha )}{1/2\rho U^{2}DL},C_{L}(\alpha )=\frac{F_{L}(\alpha )}{1/2\rho U^{2}BL},C_{M}(\alpha )=\frac{M_{z}(\alpha )}{1/2\rho U^{2}B^{2}L}$$

(13) $$C_{H}(\alpha )=\frac{F_{H}(\alpha )}{1/2\rho U^{2}DL},C_{V}(\alpha )=\frac{F_{V}(\alpha )}{1/2\rho U^{2}BL},C_{M}(\alpha )=\frac{M_{z}(\alpha )}{1/2\rho U^{2}B^{2}L}$$

where $C_{H}$, $C_{V}$ and $C_{M}$ denote drag, lift and lift-moment force coefficients in wind coordinate axis (Figure 3); D,B,L are the depth (height), width and length of the test models.

**Figure 5:**
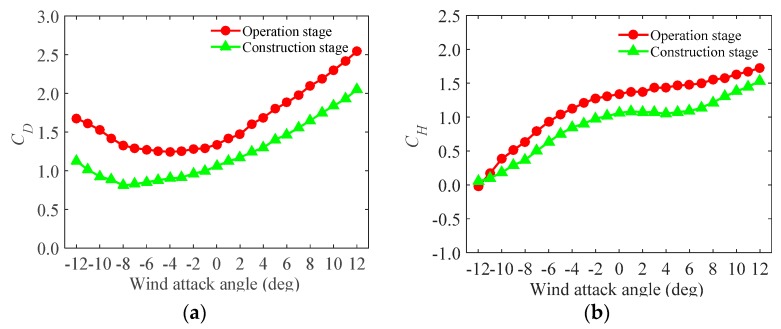
The drag force coefficients of the test models under different wind attack angles: (**a**) in local coordinate system; and (**b**) in global coordinate system.

**Figure 6:**
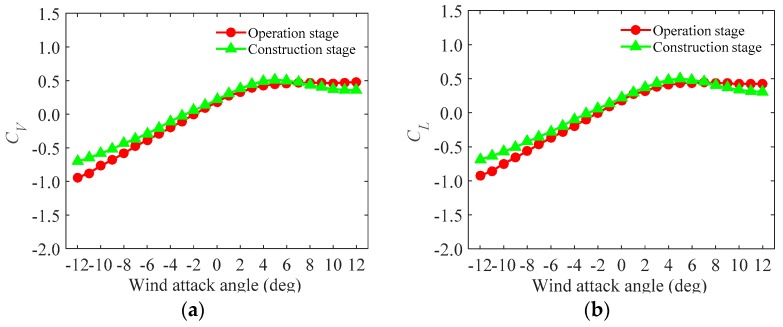
The lift force coefficients of the test models under different wind attack angles: (**a**) in local coordinate system; and (**b**) in global coordinate system.

**Figure 7:**
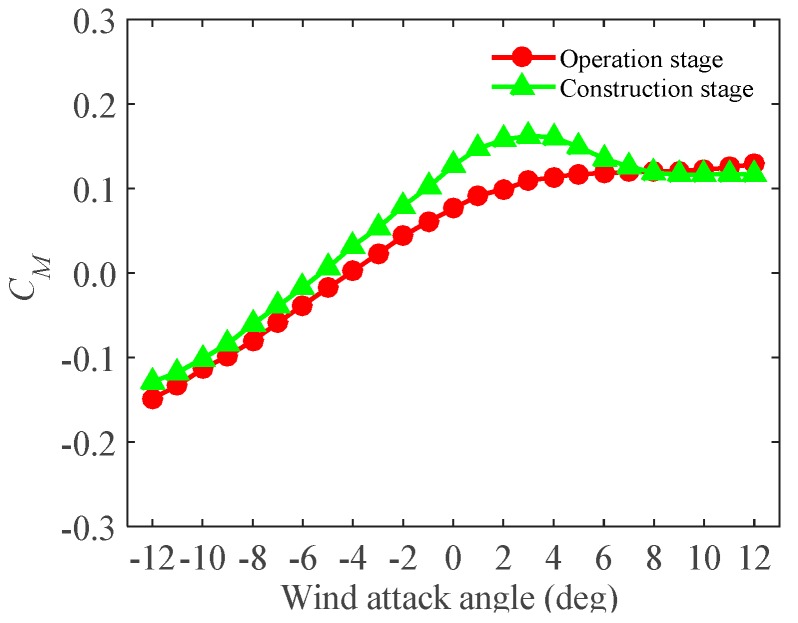
The moment force coefficients of the test models under different wind attack angles.

From the static measurement (Section 3.2), the drag, lift and moment force coefficients of the test models during the construction and the operation stages are observed and are shown in Figure 5, Figure 6 and Figure 7.

Figure 5, Figure 6 and Figure 7 show that the trends of the test models in the operation and the construction stages are in close agreement, though the magnitudes are different. The differences are induced by the effect of the accessories in the operation stage. Furthermore, Figure 5 shows that the drag force in the operation stage are larger than that in the construction stage and the maximum value occurs when the wind attack angle is $12^{\circ }$. As is known, lift and moment forces are usually much more complicated and unfavorable in the cross-wind and the torsional directions than the drag force in the along-wind directions. In the cross-wind and torsional directions, the forces may be affected by turbulence, wake excitation as well as fluid-structure interaction [25,26]. Figure 6 and Figure 7 show that, in the cross-wind and torsional directions, peaks occur at the wind attack angles of around $3^{\circ }$. Furthermore, in the directions, the force of the test model in the construction stage is larger than that in the operation stage at a specific wind attack angle range ($-12^{\circ }$ to $6^{\circ }$) and beyond this range, the force is smaller. Besides, the magnitudes of the lift and moment forces are negative and altered to be positive at the wind attack angle around $0^{\circ }$ and they are asymmetric at symmetric wind attack angle range. 

The aerodynamic forces help understand the action of wind on the bridge decks and help evaluate the aeroelastic performance of the decks (i.e., buffeting, vortex-induced vibration and flutter). The results also affirm that the wind attack angles, $\alpha =+5^{\circ },+3^{\circ },0^{\circ },-3^{\circ },-5^{\circ }$, are enough for flutter derivative and aeroelastic flutter measurements.

### 4.2. Flutter Derivatives

**Figure 8:**
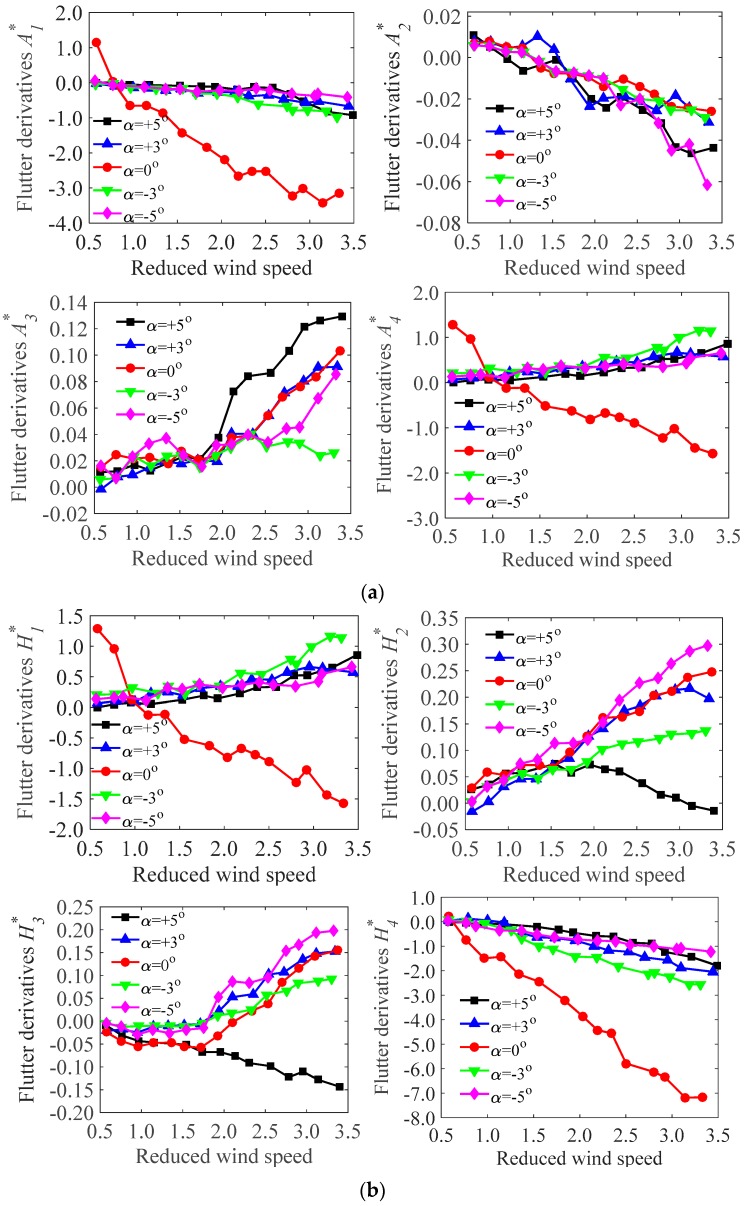
The flutter derivatives of the test model under different wind attack angles in the operation stage: (**a**) flutter derivatives $A_{1}^{*},A_{2}^{*},A_{3}^{*},A_{4}^{*}$; (**b**) flutter derivatives $H_{1}^{*},H_{2}^{*},H_{3}^{*},H_{4}^{*}$.

**Figure 9:**
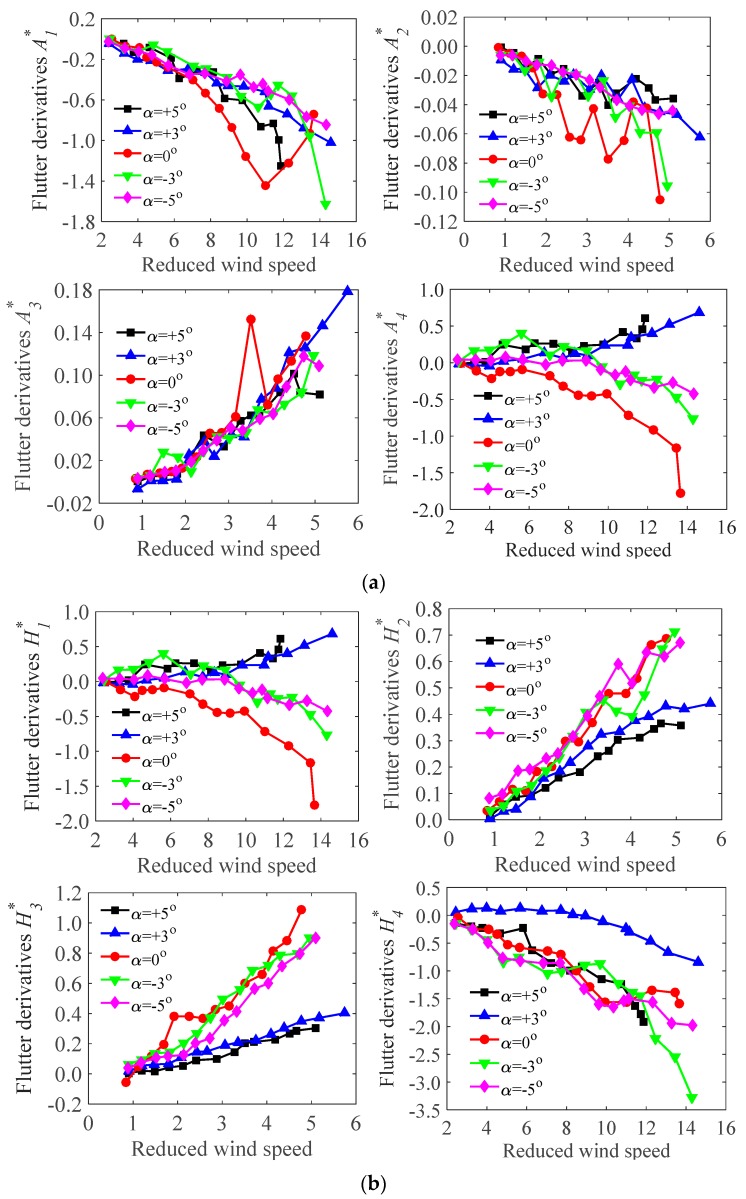
The flutter derivatives of the test model under different wind attack angles in the construction stage: (**a**) flutter derivatives $A_{1}^{*},A_{2}^{*},A_{3}^{*},A_{4}^{*}$; (**b**) flutter derivatives $H_{1}^{*},H_{2}^{*},H_{3}^{*},H_{4}^{*}$.

As mentioned, the derivatives in swaying are unimportant for the bridge deck models, and only derivatives in bending and torsion are concerned. Therefore, From the wind tunnel test (Section 3.3), the flutter derivatives of the test models during the operation and the construction stages were identified (Figure 8 and Figure 9). All the derivatives ($A_{1}^{*},A_{2}^{*},A_{3}^{*},A_{4}^{*}$; $H_{1}^{*},H_{2}^{*},H_{3}^{*},H_{4}^{*}$) under different wind attack angles ($\alpha =+5^{\circ }{,+3}^{\circ },0^{\circ },-3^{\circ },-5^{\circ }$) were obtained at a range of reduced wind speeds (*V*/*fD*, where *V* is local wind speed, *f* is oscillating frequency, and *D* is reference depth). 

It should be noted that $A_{1}^{*},A_{2}^{*}$ are velocity-dependent terms, which represent the dimensionless aerodynamic damping term for torsional vibrations. Thus, the negative values registered for $A_{1}^{*},A_{2}^{*}$implies positive aerodynamic damping acting for the torsional vibrations. $H_{1}^{*},H_{2}^{*}$ are also velocity-dependent terms, which relate to the aerodynamic damping, in the vertical vibration. $A_{3}^{*},A_{4}^{*}$ and $H_{3}^{*},H_{4}^{*}$ are amplitude-dependent terms, which relate to the aerodynamic stiffness terms in the torsional and the vertical directions. In Figure 8 (operation stage), $A_{1}^{*},\;A_{2}^{*}$ tend to decrease with wind velocities under all the wind attack angles. $H_{1}^{*},H_{2}^{*}$ tend to increase with wind velocities under all the wind attack angles except $0^{\circ }$, where $H_{1}^{*}$ decrease with increasing the wind velocities. $A_{3}^{*},A_{4}^{*}$ tend to increase with wind velocities under all the wind attack angles except $0^{\circ }$, where $A_{4}^{*}$ decreases with increasing the wind velocities. $H_{3}^{*}$ tends to increase with wind velocities under all wind attack angles except $5^{\circ }$. $H_{4}^{*}$ tends to decrease with increasing wind velocities under all wind attack angles. Similar trends of the flutter derivatives have been proven to occur at the construction stage (Figure 9). However, the magnitudes during the operation and the construction stages are different. This may be ascribed to the effect of accessories, which alter the inherent characteristics of the bridge decks. Figure 8 and Figure 9 also show that, in some cases (i.e., $\alpha {=0}^{\circ }$), $A_{1}^{*},A_{2}^{*}$ and $H_{1}^{*},H_{2}^{*}$ changes from positive to negative. This suggests that the aerodynamic damping is negative, which may induce in aerodynamic instability of test models (i.e., flutter). Besides, the flutter derivatives are varied from different wind attack angles and wind velocities. However, the trend is in close with agreement with each other though there are some deviations at isolated points. The maximum one tends to occur under the action of wind with $0^{\circ }$. The values of $A_{2}^{*},A_{3}^{*}$ have been proven to be small and very sensitive to noises. It seems that $A_{2}^{*}$ itself is unimportant and negligible. 

**Figure 10:**
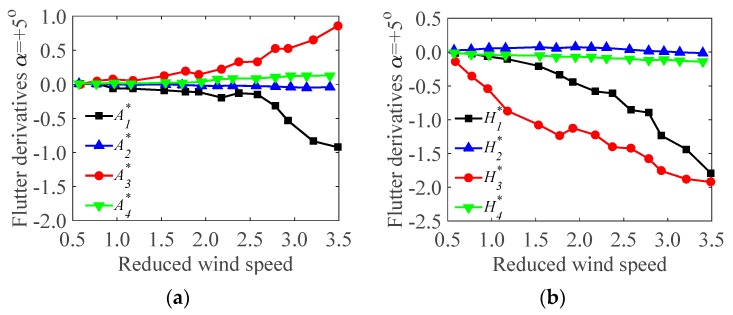
Comparisons of the flutter derivatives of the test model in the operation stage ($\alpha {=5}^{\circ }$): (**a**) flutter derivatives $A_{1}^{*},A_{2}^{*},A_{3}^{*},A_{4}^{*}$; (**b**) flutter derivatives $H_{1}^{*},H_{2}^{*},H_{3}^{*},H_{4}^{*}$.

**Figure 11:**
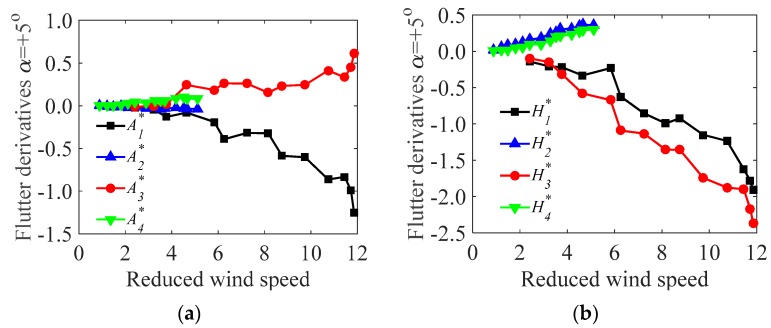
Comparisons of the flutter derivatives of the test model in the construction stage ($\alpha {=5}^{\circ }$): (**a**) flutter derivatives $A_{1}^{*},A_{2}^{*},A_{3}^{*},A_{4}^{*}$; (**b**) flutter derivatives $H_{1}^{*},H_{2}^{*},H_{3}^{*},H_{4}^{*}$.

For comparison, the flutter derivatives of the test models during the operation and the construction stages at a fixed wind attack angle ($\alpha {=5}^{\circ }$ is selected) were compared, as shown in Figure 10 and Figure 11, respectively.

In Figure 10 and Figure 11, $A_{1}^{*},A_{3}^{*}$ and $H_{1}^{*},H_{3}^{*}$ are more sensitive to the wind speed than the other derivatives, and the contribution to the aerodynamic force are significant. Furthermore, $A_{1}^{*}$ is negative and $A_{3}^{*}$ is positive under all wind velocities, and the magnitude of $A_{1}^{*}$ is larger than that of $A_{3}^{*}$. Both $H_{1}^{*}$ and $H_{3}^{*}$ are negative and tend to decrease with increasing wind velocities. Besides, Figure 10 shows that $A_{2}^{*},A_{4}^{*}$ and $H_{2}^{*},H_{4}^{*}$ are small and tend to be constant with increasing wind velocity. It seems that, in this case (the bridge deck in operation stage), the contribution of the flutter derivatives $A_{4}^{*}$ and $H_{4}^{*}$, which are related to aerodynamic stiffness terms, may be negligible. The results are partially consistent with previous studies [3,13,27], in which $A_{4}^{*}$ and $H_{4}^{*}$ were not contained in flutter derivative identifications. Similar trends have been found in the construction stage (Figure 11). 

The above results advance the understanding of the flutter derivatives of the bridge decks in the operation and the construction stages, which help to evaluate the flutter instability of the bridge decks with figuring out the critical flutter wind speeds.

### 4.3. Critical Flutter Wind Speed

The critical flutter wind speeds of the test models were determined in two ways: (1) directly measured from the aeroelastic test (Section 3.4); and (2) calculated from the observed flutter derivatives based on the method illustrated in Section 2. The critical flutter wind speeds of the test models obtained from the two ways are listed in Table 2. It should be noted that, in Table 2, the critical flutter wind speeds of the test models have been transferred to the corresponding prototypes for convenient comparison. The allowable value is determined based on the Wind-Resistant Design Specification for Highway Bridges [28]. In addition, in Table 2, some directly measured critical wind speeds are given as a range instead of a specific value. This is because, during the aeroelastic test, the measured critical wind speed is much larger than the allowable value, and the bridge is not prone to flutter under the action of wind. Therefore, it is not necessary to test at a large wind speed. 

In Table 2, the identified critical wind speeds of the test model in the operation stage under the wind attack angles of $+5^{\circ }$ and ${+3}^{\circ }$ are close to the directly measure values, and the differences are within 10%. This suggests that the critical wind speeds determined from the observed flutter derivatives are acceptable, and the derivatives are reasonable. Furthermore, the critical wind speeds in the operation stage is smaller than those in the construction stage. This indicates that the bridge deck in the operation stage is unfavorable, which may be ascribed to the effect of accessories. Besides, the minimum critical wind speed has been proven to occur at the wind attack angle of $+5^{\circ }$ and it is the most unfavorable wind attack angle. In addition, no matter the directly measured or identified wind speeds in the construction and the operation stages are much larger than the corresponding allowable values. It means that the bridge decks have good stability under the action of wind and flutters of the decks are not prone to occur.

## 5. Concluding Remarks

In this study, flutter of the Qingzhou Channel Bridge, which is an important component of the Hong Kong-Zhuhai-Macau Bridge was investigated based on wind tunnel measurements. The aerodynamic forces, flutter derivatives and flutters of the bridge decks during the construction and the operation stages were measured experimentally. Then, the results of the flutter derivatives of were discussed. From the derivatives, the critical flutter wind speeds of the bridge decks were calculated and compared with the directly measured flutters. The main conclusions are listed below.

(1) The aerodynamic forces of the test models during the operation stage and the construction stage are different. This is ascribed to the effect of accessories installed in the operation stage. The maximum lift and moment force occurs at the wind attack angle of around $3^{\circ }$.
(2) The critical wind speeds determined from the observed flutter derivative measurements are acceptable. This suggests that the observed derivatives are reasonable and the calculating method for the critical flutter wind speeds (in Section 2) is applicable.
(3) The critical wind speeds in the operation stage is smaller than those in the construction stage, which suggests that the flutter of the bridge deck in the operation stage is prone to occur. This could be ascribed to the effect of accessories in the operation stage.
(4) The directly measured or identified wind speeds in the construction and the operation stages are much larger than the corresponding allowable values, which suggests that the flutter instability of the bridge is good.

## Figures and Tables

**Table 1:** The parameters of the test models.

Parameters		Units	Scale Ratios	Prototype	Required Value	Test Value	Errors (%)
Dimensions	Height	m	1/50	4.5	0.09	0.09	0
	Width	m	1/50	38.8	0.776	0.776	0
Mass per unit length		kg/m	1/50^2^	34,754	13.092	13.89	6.1
Mass moment of inertia per unit length		kg·m^2^/m	1/50^4^	3681,159	0.589	0.586	0.5
Radius of gyration		m	1/50	10.29	0.2058	0.2054	0.07
Fundamental frequency	Bending	Hz	5.464	0.5162	2.821	2.82	0.04
	Torsion	Hz	5.476	/	/	0.32	/
Damping ratio	Bending	%	1	1.0782	5.9	5.91	0.17
	Torsion	%	1	/	/	0.32	/

**Table 2:** The critical flutter wind speeds of the test models.

Wind Attack Angles (°)	Construction Stage (m/s)			Operation Stage (m/s)		
	Directly Measured	Identified	Allowable Value	Directly Measured	Identified	Allowable Value
+5	/	275.4	66.2	102.04	92.6	79.8
+3	>140	289.3		135.45	145.7	
0	>170	398.3		>162.54	213.4	
−3	>170	411.3		>171.57	298.2	
−5	/	417.9		>171.57	356.6	
